# Tumor Necrosis Factor-α Regulates Glucocorticoid Synthesis in the Adrenal Glands of *Trypanosoma cruzi* Acutely-Infected Mice. The Role of TNF-R1

**DOI:** 10.1371/journal.pone.0063814

**Published:** 2013-05-22

**Authors:** Silvina R. Villar, M. Teresa Ronco, Rodrigo Fernández Bussy, Eduardo Roggero, Ailin Lepletier, Romina Manarin, Wilson Savino, Ana Rosa Pérez, Oscar Bottasso

**Affiliations:** 1 Instituto de Inmunología, Facultad de Ciencias Médicas, Universidad Nacional de Rosario, Rosario, Argentina; 2 Instituto de Fisiología Experimental (IFISE-CONICET), Facultad de Ciencias Bioquímicas y Farmacéuticas, Universidad Nacional de Rosario, Rosario, Argentina; 3 Departamento de Fisiología, Facultad de Medicina, Universidad Abierta Interamericana (U.A.I.), Rosario, Argentina; 4 Laboratory on Thymus Research, Oswaldo Cruz Institute, Oswaldo Cruz Foundation, Rio de Janeiro, Brazil; Albert Einstein Institute for Research and Education, Brazil

## Abstract

Adrenal steroidogenesis is under a complex regulation involving extrinsic and intrinsic adrenal factors. TNF-α is an inflammatory cytokine produced in response to tissue injury and several other stimuli. We have previously demonstrated that TNF-R1 knockout (TNF-R1^−/−^) mice have a dysregulated synthesis of glucocorticoids (GCs) during *Trypanosoma cruzi* acute infection. Since TNF-α may influence GCs production, not only through the hypothalamus-pituitary axis, but also at the adrenal level, we now investigated the role of this cytokine on the adrenal GCs production. Wild type (WT) and TNF-R1^−/−^ mice undergoing acute infection (Tc-WT and Tc-TNF-R1^−/−^ groups), displayed adrenal hyperplasia together with increased GCs levels. Notably, systemic ACTH remained unchanged in Tc-WT and Tc-TNF-R1^−/−^ compared with uninfected mice, suggesting some degree of ACTH-independence of GCs synthesis. TNF-α expression was increased within the adrenal gland from both infected mouse groups, with Tc-WT mice showing an augmented TNF-R1 expression. Tc-WT mice showed increased levels of P-p38 and P-ERK compared to uninfected WT animals, whereas Tc-TNF-R1^−/−^ mice had increased p38 and JNK phosphorylation respect to Tc-WT mice. Strikingly, adrenal NF-κB and AP-1 activation during infection was blunted in Tc-TNF-R1^−/−^ mice. The accumulation of mRNAs for steroidogenic acute regulatory protein and cytochrome P450 were significantly increased in both Tc-WT and Tc-TNF-R1^−/−^ mice; being much more augmented in the latter group, which also had remarkably increased GCs levels. TNF-α emerges as a potent modulator of steroidogenesis in adrenocortical cells during *T. cruzi* infection in which MAPK pathways, NF-κB and AP-1 seem to play a role in the adrenal synthesis of pro-inflammatory cytokines and enzymes regulating GCs synthesis. These results suggest the existence of an intrinsic immune-adrenal interaction involved in the dysregulated synthesis of GCs during murine Chagas disease.

## Introduction

The immune system plays a key role in the recognition and elimination of pathogens as well as in the regulation of the inflammatory response and tissue repair. In conjunction with the neuro-endocrine system this serves to maintain internal homeostasis [1], [2]. This immuno-endocrine cross-talk is initiated when activated immune cells release pro-inflammatory cytokines like TNF-α, IL-6 or IL-1β into the circulation, leading to activation of the hypothalamus-pituitary-adrenal (HPA) axis. HPA axis stimulation promotes the secretion of pituitary adrenocorticotropic hormone (ACTH) and adrenocortical glucocorticoids (GCs), which in turn affect the immune response [1], [2]. GCs are potent immunomodulatory agents and are well known for their anti-inflammatory effects, mainly suppressing pro-inflammatory cytokines through their antagonism with the nuclear factor-κB (NF-κB). Moreover, other transcription factors, such as the activator protein-1 (AP-1) have been shown to be crucial in inducing a number of genes involved in inflammation [3], [4].

Evidence indicates that adrenal GCs secretion can be modulated *in situ,* in an ACTH-independently fashion, by growth factors or cytokines such as TNF-α, IL-6 and IL-1β [5]–[8]. The mechanism underlying this process seems to involve a complex series of interactions in which nuclear transcription factors like NF-κB and AP-1 promote the synthesis of pro-inflammatory cytokines *in situ* that in turn modulate the expression of enzymes involved in GCs synthesis. Steroidogenesis is the main function of adrenocortical cells in the *zone fasciculata*. In this regard, studies indicate that TNF-α is able to influence the expression of steroidogenic enzymes [9], [10].

Our previous work in mice experimentally infected with *Trypanosoma cruzi* (*T. cruzi*) suggests that the immune-endocrine response accompanying the development of the infection strongly affects the progression of the disease [11], [12]. C57BL/6 mice infected with the *Tulahuen* strain of *T. cruzi* resulted in an acute fatal disease accompanied by a severe thymic atrophy and higher levels of TNF-α, compatible with an excessive inflammatory reaction arising from an unfavorable host-parasite relationship [13], [14]. Furthermore, an intense stimulation of the HPA axis was observed during infection [7], [15]. Adrenalectomy and/or blockade of receptors for GCs with the antagonist RU486 resulted in increased production of TNF-α and other pro-inflammatory cytokines in infected mice together with an accelerated death [15], [16]. Since TNF-α seems to be deleterious on the course of disease, but at the same time is involved in HPA axis activation, the relevance of TNF-α was subsequently analyzed in mice genetically deficient for the two receptors (TNF-R1^−/−^ and TNF-R2^−/−^) for this cytokine. Infected TNF-R1^−/−^ mice showed a more pronounced stimulation of the HPA axis than wild type mice [16], suggesting an imbalanced immuno-endocrine circuit.

The existence of an intra-adrenal paracrine regulation of GCs release, in which components of the immune response, like TNF-α, may play effects beyond the classical neuro-endocrine-immune interactions, provides a stimulating background for exploring its features in the context of *T. cruzi* infection. Since TNF-R1 is the form expressed in the adrenal cortex [8], we wished to analyse the role of TNF-α via TNF-R1 signalling in the regulation of GCs synthesis in the adrenal glands of *Trypanosoma cruzi* acutely-infected mice.

## Materials and Methods

### Mice, Parasite and Infection

Wild type (WT) male C57BL/6 mice and those lacking TNF-R1 (C57BL/6-*^Tnfrsf1a tm1Imx^* or TNF-R1^−/−^) originally obtained from The Jackson Laboratory and gently provided by Dr. Silvia Di Genaro, were used throughout the studies. Protocols for animal studies were approved by the Faculty of Medical Sciences Institutional Ethical Committee (Resolution N° 2003–2012). Mice were bred at the animal facilities from the School of Medicine of Rosario, had free access to food and water, and were handled according to institutional guidelines. Sixty to 90-day-old mice (3–6/group) were used in each experiment. The Tulahuén strain of *T. cruzi* employed in these studies was maintained by serial passages in C57BL/6 suckling mice. Infection was carried out by injecting 200 viable trypomastigotes of *T. cruzi* by the subcutaneous route.

### Monitoring of Acute Infection

Bloodstream forms of *T. cruzi* were assessed under standardized conditions, by direct microscopic observation of 5 µl heparinized blood obtained from the tip of the tail at different days post- infection (pi). Data are expressed as number of parasites/mL.

### Cytokine Assays

Mice were bled by cardiac puncture, and blood was collected in sterile, endotoxin-free tubes and kept refrigerated until centrifugation. Plasma was separated into small plastic vials and stored frozen at −20°C until used. Plasma cytokines were measured by specific two-site enzyme-linked immunosorbent assay (ELISA) according to the manufactureŕs specifications. ELISA kits for TNF-α, IL-1β and IL-6 (detection limits 15.6 pg/ml, 31.3 pg/ml, and 15.6 pg/ml, respectively), were purchased from Pharmingen (San Diego, CA, USA). All samples were assayed in duplicate.

### Corticosterone and ACTH Measurements

Mice were housed individually for one week before experiments started and kept single-caged throughout the experiments in temperature, and light-controlled rooms (light cycle from 7∶00 a.m. to 7∶00 p.m.). Plasma samples for hormone measurements were obtained from the tip of the tail between 8∶00 and 10∶00 a.m. as previously published [15]. Plasma CT and ACTH levels were determined by ELISA and radioimmunoassay.

### Histology

Adrenal glands were removed at day 17 post-infection (pi) and fixed in buffered formalin. In parallel, glands from uninfected animals were also processed. Five paraffin-embedded 5 µm sections from each animal were stained with haematoxylin and eosin for evaluation of morphological changes. Serial sections from the adrenals were examined in blind by an experienced pathologist. For morphometrical alterations, three areas of the largest sections in each gland were evaluated.

### Detection of TNF-R2 and Parasite Antigens by Immunofluorescence

Immunofluorescence labeling and quantitative confocal microscopy were used to investigate the distribution and quantity of TNF-R2. The presence of antigens was investigated by employing an anti *T. cruzi* serum (n = 5−6 adrenals/group). The adrenal glands were removed 17 days after infection, embedded in Tissue-Tek (Miles Inc., Elkhart, USA) and frozen in liquid nitrogen. Five 5 mm-thick cryostat sections were settled on poly- L-lysine (Sigma)-covered glass slides, acetone fixed and blocked with PBS-BSA 1%. The sections were incubated for 1 h at room temperature with one of the following primary antibodies: rat anti-mouse PE-labeled CD120b/TNF-R2 (1∶50, BD Biosciences) or anti-*T.cruzi* serum obtained from a human collection of positive sera. After 4−5 PBS rinses, polyclonal rabbit anti-human immunoglobulin G (IgG) conjugated with FITC (1∶200, DakoCytomation) was added for 1 h in the dark at room temperature. Following PBS washes, the sections were mounted in 25% glycerol/75% PBS and viewed with a laser scanning confocal microscope (Nikon eclipse TE2000-E inverted microscope, D-eclipse C1si, Melville, New York). Optimal confocal settings (aperture, gain, and laser power) were determined at the beginning of each imaging session and then held constant during the analysis of all samples. The images obtained were subsequently analyzed using the Image J software (Bethesda,Maryland, USA). The fluorescence intensity was calculated as an average of the area (i.e., the sum of the fluorescence intensity values of all pixels divided by the number of pixels in the area) and the values were recorded as arbitrary units.

### Preparation of Nuclear Extract and Determination of NF-κB and AP-1 Activation

Adrenal gland nuclear fractions were obtained by homogenization of frozen tissues in lysis buffer (PBS, 1% Triton X-100, 0.5% sodium deoxycholate, 0.1% SDS, 1 mM phenylmethylsulfonyl fluoride and 10 g/ml leupeptin). After centrifugation at 5,000 rpm for 5 min, the pellet was resuspended in the same lysis buffer and centrifuged to 5,000 rpm for 10 min again. Briefly, pellets were resuspended in buffer Hepes (20 mM Hepes, 0.4 M NaCl, 1 mM EDTA, 1 mM EGTA, 20% glycerol, protease inhibitor and dTT). Proteins were quantified by Lowry’s method [17]. Heterodimer activity in adrenal gland, NF-κB p50/p65 EZ-TFA Transcription Factor Assay (Millipore®, California, USA) was performed according to the manufacturer’s instructions. NF-κB activity assay was performed by colorimetric determination of p50 subunit in all experimental groups. AP-1 activation was tested by directly measuring c-jun from Western blots with nuclear extract. H2A (anti-histone) was used as a loading control for the nuclear fraction.

### Western Blotting

For TNF-R1, phosphoriled(P)-p38, P-ERK and P-JNK detection, tissue lysates from adrenal glands were prepared by homogenization in 3 volumes of lysating RIPA buffer containing PBS, 1% Triton X-100, 0.5% sodium deoxycholate, 0.1% SDS, 1 mM phenylmethylsulfonyl fluoride and 10 µg/ml leupeptin. After a 30-min incubation at 0°C and three freeze–thaw cycles, lysates were cleared by centrifugation at 15,000 rpm for 30 min, and supernatants were kept at −70°C. Proteins were quantified according to Lowry et al. [17]. For protein analysis, 25 µg of protein were subjected to 8% SDS-polyacrylamide gel electrophoresis and transferred to polyvinyl difluoride membranes (PVDF) (PerkinElmer Life Sciences, Boston, MA, USA). After blocking, blots were incubated overnight at 4°C with either polyclonal anti-P-p38 (1∶300, Santa Cruz Biotechnology, Santa Cruz, CA, USA) or anti-TNF-R1, anti-P-ERK, anti-P-JNK or anti-c-jun (1/1000 Cell Signalling Technologies). The membranes were then incubated with either anti-rabbit or anti-mouse IgG-peroxidase conjugates (1∶5000, Amersham Life Science) and the resulting bands were detected by enhanced chemiluminescence detection (ECL; Pierce western blotting substrate). Autoradiographs were obtained by exposing PVDF membranes to Kodak XAR film and the bands were quantified by densitometry (Shimadzu CS-9000).

### Expression of GCs Biosynthesis/activation Enzymes and Inflammatory Cytokines in the Adrenals

For the evaluation of gene expression, RNA was isolated from adrenals using TRIzol (Invitrogen, Life Technologies), according to the manufacturer’s instructions. RNA quality and quantity were assessed using an Agilent bioanalyzer (Caliper Technologies Corp., Massachusetts, USA). First strand cDNA synthesis was prepared with 1.0 µg total RNA, oligo primer, using the SuperScript TM III Reverse Transcriptase (Invitrogen, Life Technologies). Real-time PCR was performed with approximately 50 ng of cDNA for each sample and Fast SYBR Green Master Mix (Applied Biosystems, California, USA). cDNA was amplified using specific murine primer sequences reported in Table 1. All reactions were run on the Step one Plus Real-Time PCR System instrument (Applied Biosystems), according to the manufacturer’s instructions. The amplification program included an initial denaturation step at 95°C for 10 min, followed by 40 cycles of denaturation at 95°C for 15 s and annealing and extension at 60°C for 1 min. The relative gene expression of IL-1β, TNF-α, IL-6, StAR, CYP11A1, CYP11B1, 11β-HSD1, 11β-HSD2 and kDNA from *T.cruzi* was assessed by comparing the expression of each element with the normalizer RPL13 using the Ct method (2^-ΔCt^x 1,000) as previously described [18]. Green fluorescence was measured after each extension step, and the specificity of amplification was evaluated by melting curve analysis. Every sample was run in three parallel reactions.

**Table 1:** Primer sequences and expected amplification products.

Gene		Primer sequence (5′-3′)
**RPL13a**	FW	CCAAGCAGGTACTTCTGGGCCGGAA
	RV	CAGTGCGCCAGAAAATGCGGC
**TNF-α**	FW	TGTCTACTGAACTTCGGGGT
	RV	TCCACTTGGTGGTTTGCTAC
**IL-6**	FW	CTCTGCAAGAGACTTCCATCC
	RV	AGGTCTGTTGGGAGTGGTAT
**IL-1β**	FW	AGCTTCCTTGTGCAAGTGTC
	RV	CCCTTCATCTTTTGGGGTCC
**CYP11A1**	FW	GACCTGGAAGGACCATGCA
	RV	TGGG TGTACTCATCAGCTTTATTGA
**CYP11B1**	FW	TCAGTCCAGTGTGTTCAACTATACCA
	RV	GCCGCTCCCCAAAAAGA
**StAR**	FW	TCACTTGGCTGCTCAGTATTGAC
	RV	GCGATAGGACCTGGTTGATGA
**11β -HSD1**	FW	TGGTGCTCTTCCTGGCCTACT
	RV	CTGGCCCCAGTGACAATCA
**11β-HSD2**	FW	CCGTGTTCTGGAAATCACCAA
	RV	AATATTGAGGCCAGCGTTGTTAA
***T.cruzi-*** ** kDNA**	FW	AAATAATGTACGGG(G/T)GAGATGCATGA
	RW	GGTTCGATTGGGGTTGGTGTAATATA

RPL13a, ribosomal protein L13a; StAR, steroidogenic acute regulatory protein; CYP11A1, cytochrome P450, family 11, subfamily A, polypeptide1; CYP11B1, cytochrome P450, family 11, subfamily B, polypeptide1; 11β-HSD1, 11β-hydroxysteroid dehydrogenase type 1, 11β-HSD2, 11β-hydroxysteroid dehydrogenase type 2 and kDNA from *T. cruzi*.

### Statistical Analysis

Data are presented as mean ± standard error (SEM), unless otherwise stated. Statistical analysis was performed by the non parametric analysis of variance followed by Dunn post-hoc comparison when applicable. Correlation analysis was carried out using Spearman correlation test. The GraphPad Instat 4.0 software (GraphPad, California, USA) was applied for statistical analyses, and differences were considered significant was when *p* value was <0.05.

## Results

Based on data recorded in an earlier report [16], we decided to carry out present studies on day 17 pi for two main reasons. Mainly because TNF-R1^−/−^ infected mice start to die around during the 3^rd^ week of infection and between-group differences during the first two weeks pi are not well flourished but become quite noticeable at day 17 pi.

### Monitoring of Acute Infection, TNF-R1 Expression in the Adrenal Gland and Levels of Circulating Cytokines

**Figure 1:**
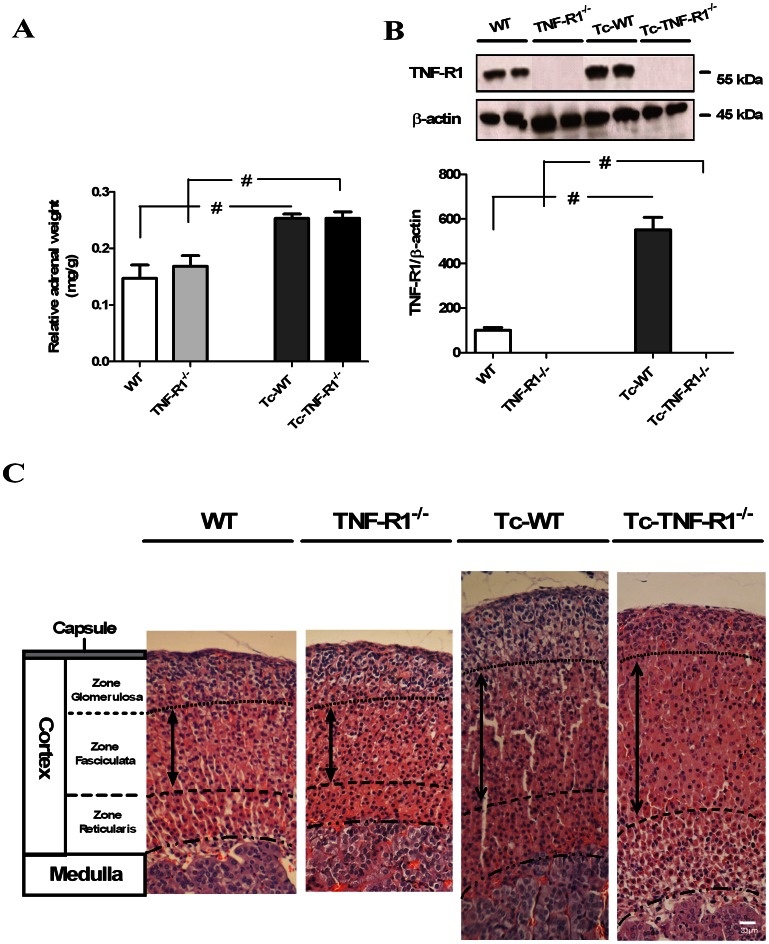
Changes in relative weight, TNF-R1 expression and morphology in endocrine glands from *T. cruzi*-infected mice. The relative weight of the adrenal glands in C57BL/6 acutely infected with *T. cruzi*, at day 17 pi (panel **A**) was calculated as follows: [adrenal weight (mg)/body weight (g)]. Panel **B** shows western blots for the protein expression of TNF-R1 at day 17 pi. Results are expressed as mean ± SEM from 3–5 mice/group. A representative experiment from 2 independent series is shown. # p<0.05 vs. uninfected counterparts. Panel **C**. The cortex consists of three morphologically distinct zones. The region immediately beneath the capsule is called *zone glomerulosa* and constitutes about 1/5 or less of the entire thickness, this zone was only increased in Tc-WT mice. The mid portion of the cortex, called *zone fasciculata*, comprises about 50–65% of the thickness of the cortex in uninfected mice (WT and TNF-R1^−/−^ mice), with Tc-WT and Tc-TNF-R1^−/−^ mice showing a diffuse hyperplasia (the broad arrow indicates the thickness with an arbitrary vertical line). The most inner portion of the cortex, called *zone reticularis*, comprises about 20–25% of the cortex from uninfected mice, showing a hyperplasia in Tc-TNF-R1^−/−^ mice. H&E stain, magnification 40 X. Scale bar: 20 µm. WT (wild type) and TNF-R1^−/−^ (TNF receptor deficient) control mice; Tc-WT and Tc-TNF-R1^−/−^ infected counterparts.

As shown previously (16), mortality was 100% in TNF-R1^−/−^ and wild type mice, but their survival time was different, being significantly shortened in infected TNF-R1^−/−^ mice, when compared to Tc-WT mice [Mean survival time in days: Tc-TNF-R1^−/−^18±0,5; Tc-WT 25±1 p<0.05]. Moreover, parasitological studies at day 17 pi revealed that Tc-TNF-R1^−/−^ mice exhibited higher parasitemias [median (rank): 24150 (36800-20470)] than Tc-WT [6670 (10695-4140), p<0.01]. Studies at the same time point indicated that the relative adrenal weight in Tc-TNF-R1^−/−^ and Tc-WT mice was significantly higher than their uninfected counterparts (p<0.05, **Figure 1A**).

Western blot analysis showed a constitutive expression of TNF-R1 in adrenal cells from WT mice, which was increased after infection (**Figure 1B**). As expected, the TNF-R1 protein was absent in TNF-R1^−/−^ mice. Plasma levels of TNF-α, IL-1β and IL-6 in Tc-TNF-R1^−/−^ were 5.1-, 4.5 and 1.16-fold increased respectively, when compared to the Tc-WT group (**Table 2**).

**Table 2:** Cytokine levels in plasma of mice undergoing acute *T. cruzi* infection.

Plasma levels(pg/mL)	WT	TNF-R1^−/−^	Tc-WT	Tc-TNF-R1^−/−^
TNF-α	nd	nd	588±99*	2999±324^&, #^
IL-6	8.5±5.1	55.1±7.1*	528±68*	612±81^&^
IL-1β	22.7±4.5	89.8±10.8*	82.1±15.7*	370.8±73.9^&, #^

* p<0.05 vs WT; & p<0.05 vs TNF-R1^−/−^; # p<0.05 vs Tc-WT; nd: non detectable.

Values represent means ± sem of five mice/group, at 17 days p.i.

WT (wild type) and TNF-R1^−/−^ (TNF receptor deficient) control mice; Tc and Tc TNF-R1^−/−^ infected counterparts.

A representative experiment from 2 independent series is shown.

The morphometrical analysis was performed to assess the size of different zones in adrenal cortex. Values (pixel^2^) depicted in **Table 3** indicate that in both infected groups, adrenal glands were significantly larger that in uninfected counterparts. This increase was partly due to the enlargement of the zone fasciculate, the most prominent in steroid-producing adrenal cells. Weight and size of the gland were clearly associated.

**Table 3:** Morphometric analysis of adrenal sections of mice undergoing acute *T. cruzi* infection.

	WT	TNF-R1^−/−^	Tc-WT	Tc-TNF-R1^−/−^
Cortex surface (pixel^2^)	371642±10382	401614±19697	821799±35461*	814334±29811^&^
Zone Glomerulosa (pixel^2^)	80863±2964	84724±1717	214601±15361*	165483±3873^&,#^
Zone Fasciculata (pixel^2^)	168449±4706	189758±12629	340718±20565*	383594±23468^&^
Zone Reticularis (pixel^2^)	131846±4086	132721±7253	283326±38567*	268790±3632

* p<0.05 vs WT; & p<0.05 vs TNF-R1^−/−^; # p<0.05 vs Tc-WT.

Values represent means ± sem of six mice/group, at 17 days p.i.

WT (wild type) and TNF-R1^−/−^ (TNF receptor deficient) control mice; Tc and Tc TNF-R1^−/−^ infected counterparts.

A representative experiment from 2 independent series is shown.

### Increase in Plasma Levels of Corticosterone without Changes in ACTH Levels during Acute *T. cruzi* Infection

**Figure 2:**
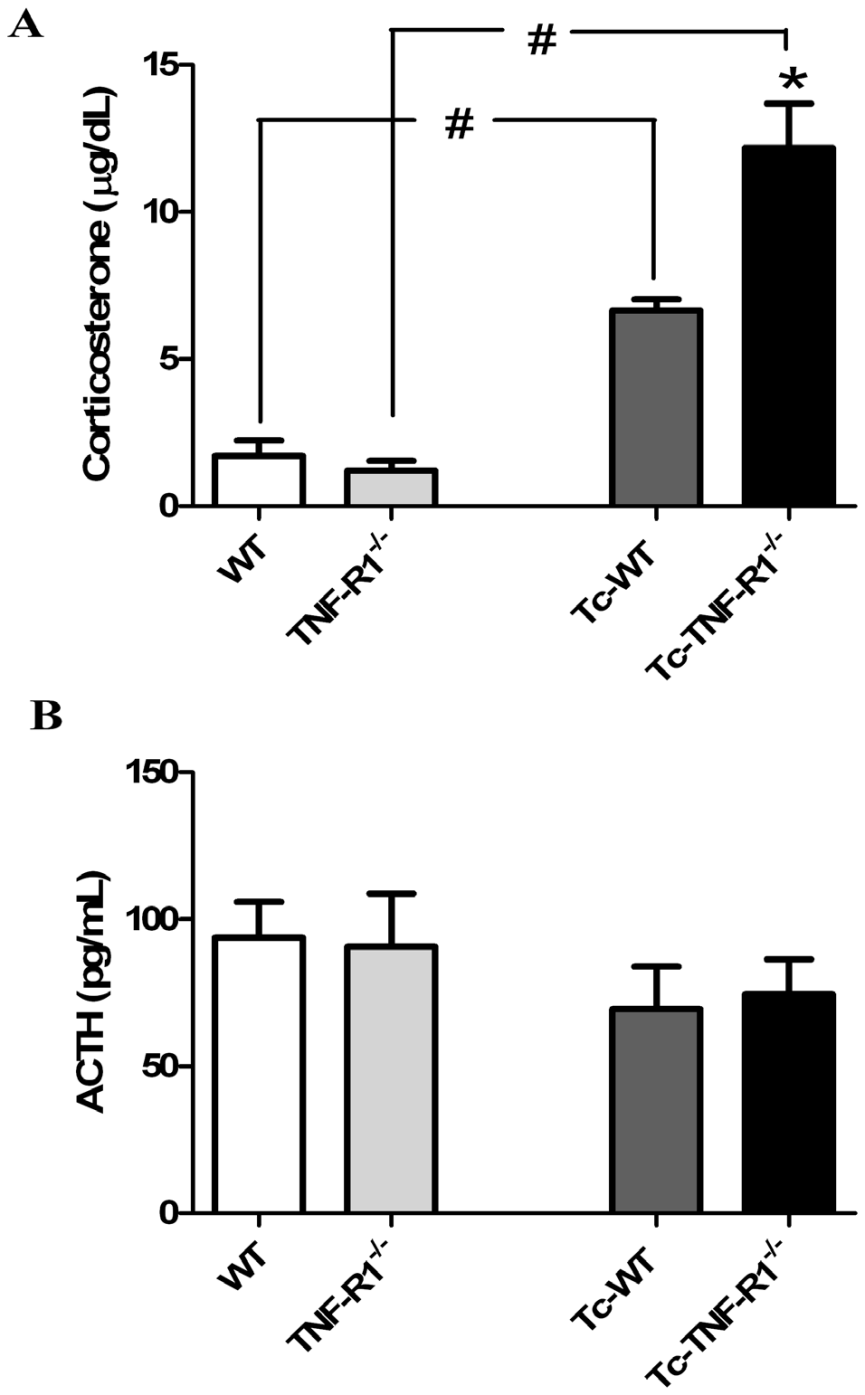
Hormonal variations in the HPA axis from *T. cruzi*-infected mice. Plasma levels of corticosterone (µg/dl, panel **A**) and ACTH (pg/ml, panel **B**) in C57BL/6 mice acutely infected with *T. cruzi*. Results are expressed as mean ± SEM from 3–5 mice/group. A representative experiment from 2 independent series is shown. WT (wild type) and TNF-R1^−/−^ (TNF receptor deficient) control mice; Tc-WT and Tc-TNF-R1^−/−^ infected counterparts. *p<0.05 vs. Tc-WT; #p<0.05 vs. uninfected counterparts.

To assess whether the increase of systemic pro-inflammatory cytokine levels from infected mice was accompanied by changes in the HPA axis, levels of CT and ACTH were measured. Uninfected mice (WT and TNF-R1^−/−^ mice) showed no significant differences in the basal levels of CT. In contrast, CT concentrations in Tc-TNF-R1^−/−^ mice [12.47±1.95 µg/dl] were 2-fold higher (p*<*0.01) than those observed in Tc-WT mice [6.73±0.45 µg/dl; **Figure 2A**]. However, plasma ACTH levels were similar in both groups (**Figure 2B**). Correlation analysis revealed no association between CT and ACTH [r_s_ = 0.139, n = 20, ns], suggesting a certain degree of ACTH-independency of GCs production. Increase in CT levels during infection in both groups of mice was accompanied by marked morphological alterations in the adrenal cortex. In line with morphometrical studies, both infected group had an increased cortical thickness, largely because of an enlarged *zone fasciculata*. In Tc-WT, the *zone glomerulosa* was also increased with polyhedral non-vacuolated eosinophilic cells, whereas the *zone reticularis* showed a diffuse hyperplasia. In the central cortical area some nodular hyperplasia was also seen. Conversely, no hyperplasia of the *zone glomerulosa* was seen in TNF-R1^−/−^ mice, showing a remarkable hypertrophy of the *zone reticularis*. No parasite nests were observed. Adrenal glands from uninfected mice had a normal morphology (**Figure 1C** **)**.

### NF-κB and AP-1 Activation in the Adrenals during *T. cruzi* Infection is Blunted in Absence of TNF-R1

**Figure 3:**
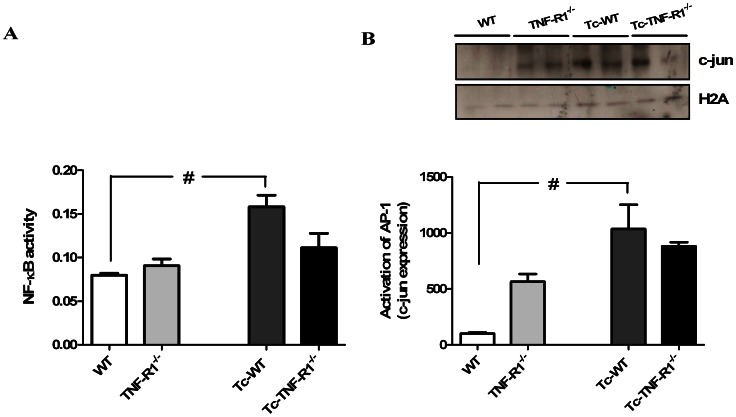
Activation of NF-κB and AP-1 in adrenal gland during *T. cruzi* infection. Levels of NF-κB activity (OD 450/650, panel **A**) and activation of AP-1 (panel **B**) in nuclear extracts of adrenal glands from C57BL/6 mice acutely infected with *T. cruzi*. NF-κB activity assay was assessed by colorimetric determination of p50 subunit in all experimental groups. AP-1 activation was tested using direct measurement of c-jun by Western blot. Results are shown as mean ± SEM from 3–5 mice/group/day. A representative experiment from two similar rounds is shown. WT (wild type) and TNF-R1^−/−^ (TNF receptor deficient) control mice; Tc-WT and Tc-TNF-R1^−/−^ infected counterparts. #p<0.05 vs. uninfected counterparts; &p<0.05 vs. WT mice.

Transcription factors NF-*κ*B and AP-1 are key components of TNF-α signal transduction pathway. Moreover, it is known that TNF-α can activate NF-κB through the TNF-R1-mediated signaling. To evaluate the NF-κB activity in the adrenal glands during *T. cruzi* infection a nuclear fraction was prepared. As shown in **Figure 3** there was a strong induction of NF-κB activation in adrenal cells obtained from Tc-WT mice. Interestingly, NF-κB activity in Tc-TNF-R1^−/−^ and TNF-R1^−/−^ mice remained within the values seen in the control group (**Figure 3A**), suggesting a key role of TNF-R1 in NF-κB activity during infection.

AP-1 is a collective term referring to dimeric transcription factors composed of c-Jun and c-Fos subunits that bind to a common DNA site, the AP-1 binding site. We investigated the activation of AP-1 through direct measurement of c-jun. Western blotting of nuclear extracts revealed increased levels of c-jun in both groups of infected mice compared to their uninfected counterparts (**Figure 3B**). Noteworthy, uninfected TNF-R1^−/−^ animals exhibited higher basal levels of this protein in the nuclear fraction respect to WT mice, for which the relative increment in c-jun after infection became more marked in WT mice. The relative increase in c-jun activation was calculated according the following formula: values in *T. cruzi*-infected mice/uninfected mice. [Relative change, means ± SEM: WT = 10.1±1.1, TNF-R1^−/−^ = 1.5±0.9, p<0.05].

### TNF-R1 Deficiency Results in a Differential Level of MAPK Phosphorylation in Response to *T. cruzi* Infection

**Figure 4:**
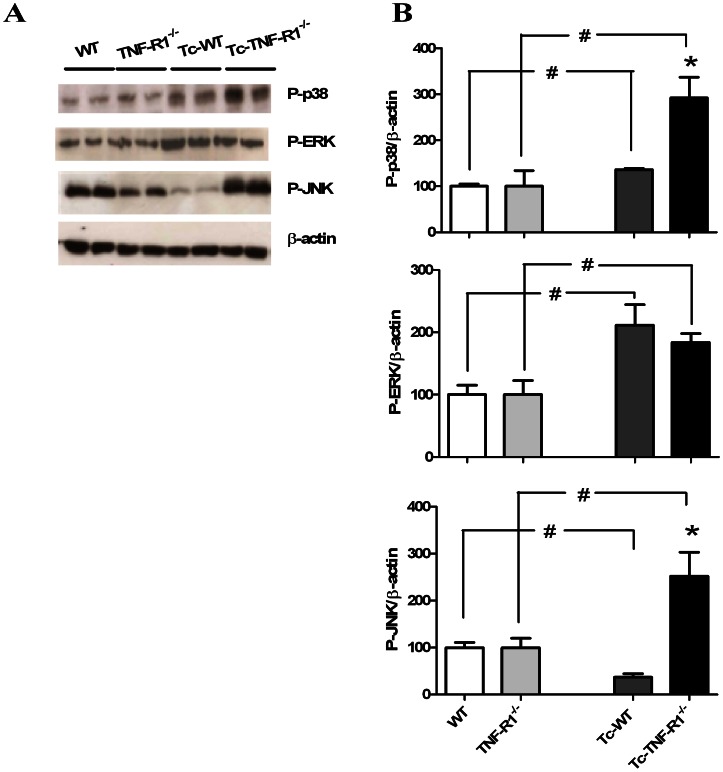
Activation of MAPK pathways in adrenal glands from mice acutely infected with *T.cruzi *. Blots correspond to P-p38, P-ERK and P-JNK. Blots were stripped and revealed with an anti-β-actin (panel **A**). Bars represent the densitometry considering WT and TNF-R1^−/−^ as 100% (panel **B**). Expression is relative to β-actin. Results are expressed as mean ± SEM from 3–5 mice/group. Data correspond to one round of two representative and independent experimental rounds. WT (wild type) and TNF-R1^−/−^ (TNF receptor deficient) control mice; Tc-WT and Tc-TNF-R1^−/−^ infected counterparts. *p<0.05 vs. Tc-WT; #p<0.05 vs. uninfected counterparts.

TNF-α may lead to the activation of various MAPK signaling networks, contributing to the induction of NF-κB and AP-1 nuclear factors implicated in the synthesis of pro-inflammatory cytokines and expression of steroidogenic enzymes. Results presented in **Figure 4A–B** demonstrate an increase in the amount of P-p38 and P-ERK in adrenal cell homogenates from Tc-WT mice compared with control animals; whereas the amount of P-JNK was reduced (p<0.05). As depicted in the same figure, Tc-TNF-R1^−/−^ mice had a higher expression of P-p38, P-ERK and P-JNK proteins as compared to their uninfected counterparts, together with an increased expression of P-p38 and P-JNK in comparison with Tc-WT mice (p<0.05). These results indicate that TNF-R1 deficiency during *T. cruzi* infection enhances MAPK activation in adrenal cells.

### Increase of Glucocorticoid-related Gene Expression in the Adrenal Glands of *T. cruzi* Acutely-infected Mice

**Figure 5:**
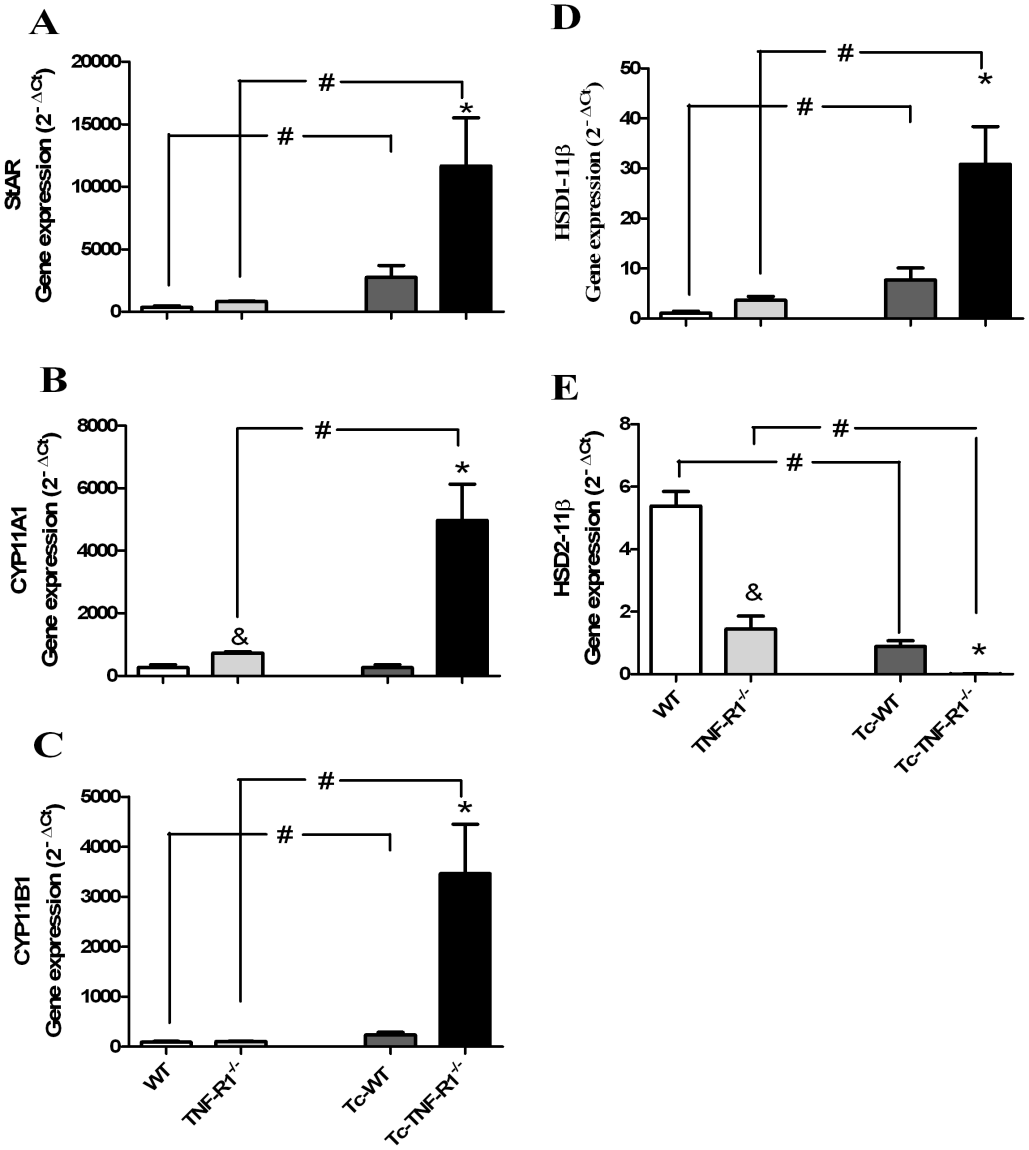
Steroidogenic enzymes gene expression in adrenal glands from *T. cruzi* infection in absence of TNF-R1. Expression profiles of genes involved in GC synthesis/activation enzymes, StAR (panel **A**), CYP11A1 (panel **B**), CYP11B1 (panel **C**), 11β-HSD1 (panel **D**) and 11β-HSD2 (panel **E**) in adrenal glands from C57BL/6 or TNF-R1^−/−^ mice acutely infected with *T. cruzi*. Total mRNA was purified and analyzed by Real-Time-PCR using nonsaturating conditions. The mRNA expression levels were then normalized to RPL13. Results are shown as mean ± SEM from 3–4 mice/group/day. A representative experiment from 3 independent series is shown. WT (wild type) and TNF-R1^−/−^ (TNF receptor deficient) control mice; Tc-WT and Tc-TNF-R1^−/−^ infected counterparts. *p<0.05 vs. Tc-WT; #p<0.05 vs. uninfected counterparts and &p<0.05 vs. WT.

Genes encoding for key steroidogenic enzymes like acute regulatory protein (StAR), cytochrome P450 enzymes like family 11 (subfamilies A and B, polypeptide 1 -CYP11A1 and CYP11B1, respectively) are involved in *de novo* GCs synthesis. Interestingly, as seen in **Figure 5**, mRNA expression levels for StAR and CYP11B1 enzymes were significantly increased in both Tc-WT and Tc-TNF-R1^−/−^ mice; much more augmented in the latter group. By opposite, mRNA expression levels of CYP11A1 remained unchanged in Tc-WT, but were significantly increased in Tc-TNF-R1^−/−^ group.

The activity of GCs also depends on 11β-HSDs. The endogenous 11-dehydrocorticosterone is inactive and require conversion to their active 11β-hydroxy derivative CT. Type 1 11β-HSD1 catalyzes this conversion, whereas, 11β-HSD2 breaks down natural GCs as they enter the cell resulting in inactive metabolites [19]. As such, changes in the relative activity of 11 β-HSDs may influence the intracellular availability of GCs and impact on immune responses. When exploring the expression of the gene encoding for 11β-HSD1 enzyme, its mRNA expression was increased in Tc-WT group, and dramatically elevated in Tc-TNF-R1^−/−^ (4- and 7-fold higher than the levels seen in non infected WT and TNF-R1^−/−^ mice, respectively **Figure 5D–E**). By contrast, a decreased expression of the 11β-HSD2 gene was found in both groups of infected mice.

### Preferential Expression of Pro-inflammatory Cytokine-related Genes in the Adrenals from Infected TNF-R1^−/−^ Mice

**Figure 6:**
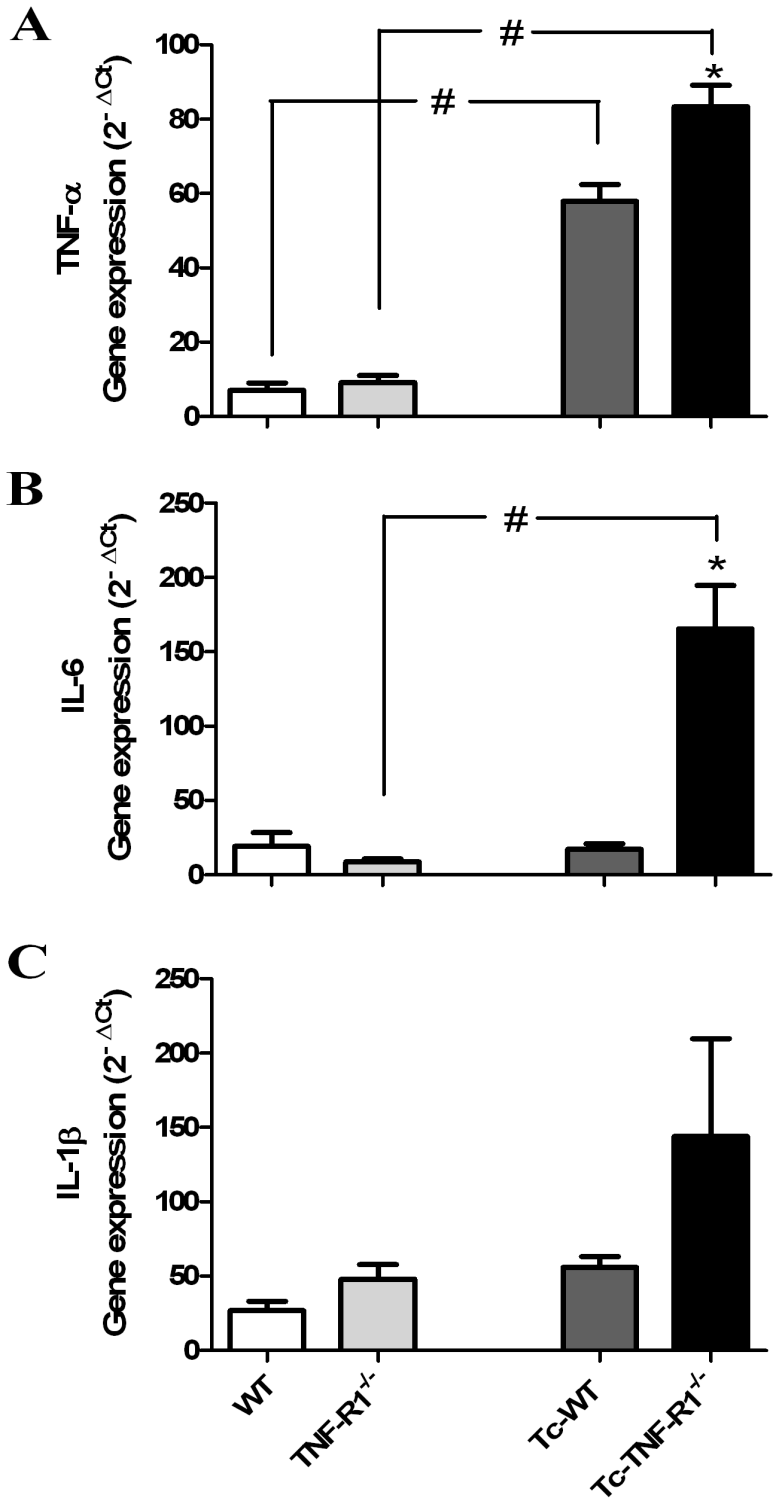
Gene expression of pro-inflammatory cytokines in adrenal glands from *T. cruzi*-infected mice. Total RNA was extracted and the mRNA levels of TNF-α (panel **A**), IL-6 (panel **B**) and IL-1β (panel **C**) were assessed by quantitative PCR (normalized to RPL13). Results are shown as mean ± SEM from 3–4 mice/group/day. A representative experiment from 3 independent series is shown. WT (wild type) and TNF-R1^−/−^ (TNF receptor deficient) control mice; Tc-WT and Tc- TNF-R1^−/−^ infected counterparts. *p<0.05 vs. Tc-WT; #p<0.05 vs. uninfected counterparts.

*In vitro* studies demonstrated that in response to external stimuli, adrenal cells synthesize and release IL-6 and TNF-α [8], [20]. It follows that under various physiological and pathological conditions, the *in situ* cytokine profile may be differentially regulated, for which the expression of cytokine-related genes was analyzed. **Figure 6A** shows that during the infection there was an increase in TNF-α gene expression levels in both groups of infected mice. In contrast, the expression level of IL-6 mRNA remained unchanged in Tc-WT but was significantly increased in Tc-TNF-R1^−/−^ group (**Figure 6B**). When analyzing IL-1β mRNA, an increased but statistically insignificant gene expression in Tc-TNF-R1^−/−^ mice was found (**Figure 6C**).

### Evaluation of TNF-R2 Expression and the Presence of Parasite Antigens

**Figure 7:**
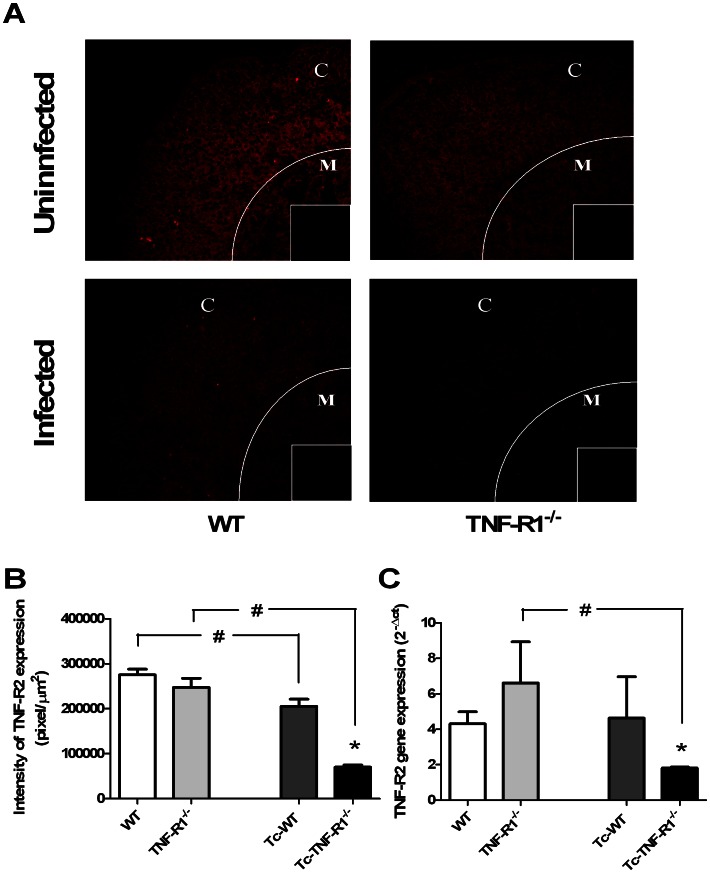
Expression of TNF receptor type 2 in adrenal gland from *T. cruzi-* infected mice. Panel **A** shows frozen sections of the adrenal gland labeled with anti-TNF-R2 antibody. Confocal microscopy shows a decreased in TNF-R2 contents in cortex of the adrenal gland in Tc-WT and Tc-TNF-R1^−/−^ (more pronounced in Tc-TNF-R1^−/−^ group) after 17 days of infection. The small box is a representative control staining in which an unrelated primary antibody was applied. Panel **B** shows graphs corresponding to the relative quantification analysis of cortical TNF-R2 expression of 5–6 microscopic fields of adrenal gland from WT (wild type) and TNF-R1^−/−^ (TNF receptor deficient) control mice and infected counterparts (Tc-WT and Tc-TNF-R1^−/−^). Panel **C** shows the gene expression of TNF-R2 in adrenal glands from *T. cruzi*-infected mice. Results are expressed as mean ± SEM. A representative experiment from 2 independent series is shown. The scale bar represents 50 µm (C: cortex; M: medulla). Original magnifications 200X. * p<0.05 vs. Tc-WT; # p<0.05 vs. uninfected counterparts.

To discard the potential contribution of TNF-R2 or the parasite presence in the activation pathways, their expression in adrenal gland was analyzed by using immunofluorescence labeling and real time (**Figure 7** **and** **8**). Remarkably, the fluorescent intensity for TNF-R2 was significantly decreased in Tc-WT and Tc-TNF-R1^−/−^ mice compared to their uninfected counterparts (**Figure 7A–B**). Furthermore, the Tc-TNF-R1^−/−^ group had a much less expression of TNF-R2 than Tc-WT (**Figure 7C**). Both results suggested that TNF-R2 signaling pathway is not influential during infection.

**Figure 8:**
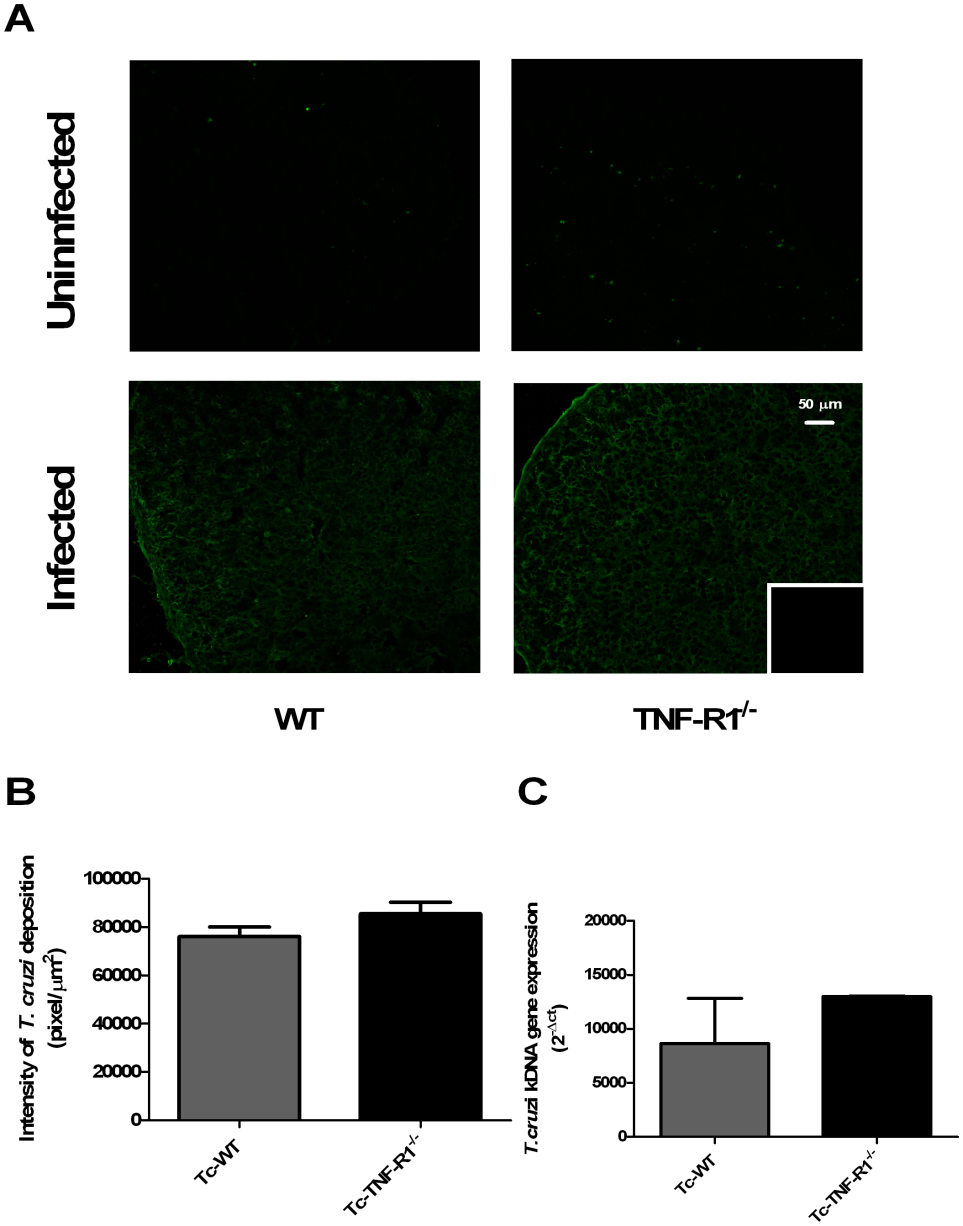
Detection of *T. cruzi* in the adrenal gland. Panel **A** shows frozen sections of the adrenal gland labeled with anti–*T. cruzi* antiserum. Immunostaining analysis demonstrated alike deposition of parasite antigens in the adrenal glands from Tc-WT and Tc-TNF-R1^−/−^ after 17 days of infection. The small boxes represent a control staining in which an unrelated primary antibody was applied. Panel **B** corresponds to the relative quantification analysis of cortical *T.cruzi*-labeling of 5–6 microscopic fields of adrenal glands from WT (wild type) and TNF-R1^−/−^ (TNF receptor-1 deficient) control mice and their infected counterparts (Tc-WT and Tc-TNF-R1^−/−^). Panel **C** shows the quantitative expression of *T. cruzi* kDNA in adrenal glands from *T. cruzi*-infected mice. Results are expressed as mean ± SEM. A representative experiment from 2 independent series is shown. The scale bar represents 50 µm. Original magnifications 200X.

Immunofluorescence assay with anti–*T. cruzi* antiserum revealed a similar deposition of parasite antigens in the adrenals from both groups of *T. cruzi*-infected mice, while the search for the presence of amastigote nests was not successful (**Figure 8A–B**). Nevertheless, through the use of a highly sensitive technique (RT-PCR for parasite-specific kinetoplast-related genes), *T. cruzi*-kDNA was detected at similar levels in adrenal glands from both infected groups, revealing a very low presence of parasites (**Figure 8C**).

The bulk of these results suggest that differences observed in transcription pathways in adrenal glands from Tc-TNF-R1^−/−^ mice compared to Tc-WT are not linked to differences, at least, in the TNF-R2 or parasite load.

## Discussion

The adrenal gland plays a critical role in the host’s response to immune stress, as it constitutes the fundamental source of GCs production [21]. Activation of the adrenal cortex by immune challenge occurs via hormone release by the hypothalamus and pituitary gland [22], [23]. Nevertheless, there is also evidence that inflammatory messengers like cytokines, such as IL-1, IL-6 and TNF-α, influence the release of this stress hormone through direct effects on the adrenal cells [8], [19]. Hence, inflammatory cytokines are capable of maintaining a high GCs secretion, not only through neuro-endocrine mechanisms but also via an immune-endocrine process within the adrenal gland [24], [25].

At first sight, one may assume that the adrenal hyperplasia seen during *T. cruzi* infection is the sole result of the HPA axis activation mediated by pro-inflammatory cytokines. The dissociation between ACTH plasma concentrations, which remained unchanged in *T. cruzi*-infected mice, and increased levels of CT, suggests that factors acting at the adrenal level may be also accounting for this finding. Our results point out that an intra-adrenal cytokine network seems to be involved in the modulation of GCs release. Compared to *T. cruzi*-infected mice, infected TNFR1^−/−^ counterparts, which presented higher CT circulating levels, had an increased intra-adrenal expression of IL-1β, IL-6 and TNF-α; whereas only TNF-α was increased in Tc-WT mice. These findings fit well with *in vitro* studies showing that cytokines can directly affect GCs release by the adrenal gland, wherein IL-6 and IL-1 exert a stimulatory effect, whereas TNF-α effect may be rather inhibitory [8], [26]–[28].

Moreover, it was demonstrated that TNF-α inhibits basal cortisol release from bovine *zone fasciculata* adrenal cells [19]. In addition, TNF-α has been shown to inhibit the ACTH-induced increase in cytochrome P450 oxidase mRNA expression (a group of enzymes involved in GC synthesis) in cultured human fetal adrenal cells [10]. However, TNF-α promotes an increase of cortisol release from adult human adrenal cells *in vitro* [29]. These contradictory data leave the significance of TNF-α in the regulation of adrenal function unclear. The direct effect of TNF-α on the adrenal gland contradicts the stimulatory effect of circulating TNF-α on hypothalamus and pituitary gland activity seen in an intact animal. Therefore, the role of intra-adrenal TNF-α may be different from that of circulating TNF-α.

This cytokine mediates the inflammatory response mainly via the TNF-R1 expressed by various cell types, including lymphocytes and macrophages. In WT mice, TNF-R1 was expressed in the adrenal glands under basal conditions, markedly increasing during *T. cruzi* infection. This finding was accompanied by a strong induction of NF-κB activation, not seen in Tc-TNF-R1^−/−^ mice. In the nucleus, NF-κB binds to target DNA elements and positively regulates the transcription of genes involved in immune and inflammatory responses, enzyme expression, cell growth control, and apoptosis. The fact that Tc-WT mice showed a significantly increased NF-kB activity (whereas Tc-TNFR1^−/−^ did not) suggests that changes in cytokine and GCs production in the latter group are mediated by a different signaling pathway. The lower TNF-R2 expression is suggestive of a non significant role of this signaling pathway. In present studies, infection with *T. cruzi* does lead to the activation of NF-κB and different MAPK cascades within the adrenal gland. Besides NF-κB, MAPK have been implicated in the synthesis of pro-inflammatory cytokines, acting as positive regulator for the expression of a variety of genes such as TNF-α, IL-6 and other inducible enzymes i.e., the ones involved in GCs production [30]–[33]. As regards Tc-WT mice, they had an increased p-38 MAPK and p-ERK expression, with p-JNK expression being found decreased in them. In turn, Tc-TNF-R1^−/−^ mice showed a remarkably increased p-JNK and p-38 MAPK expression together with an augmented p-ERK expression as seen in the Tc-WT group. Evidence indicates that p-JNK is associated to other transcription factors, like AP-1, positively influencing the synthesis of pro-inflammatory cytokines and GCs [31], [34]. This may partly account for the inhibitory effect of TNF-α in the synthesis of GCs by the adrenal gland. Interestingly, uninfected TNF-R1^−/−^ mice had increased levels of IL-6 respect WT. Therefore, the higher serum CT levels observed in Tc-TNF-R1^−/−^ mice compared with Tc-WT mice may result both from a direct negative effect of TNF-α on adrenal CT synthesis, together with the non mutually excluding influence of increased IL-6 levels, known to up-regulate adrenal GCs synthesis.

In line with the increased plasma levels of CT, high expression level of GCs-related *de novo* synthesizing enzymes were found in both groups of *T. cruzi*-infected mice. Also, an even higher expression of enzymes driving GCs secretion was seen in Tc-TNF-R1^−/−^ mice consistent with their more augmented levels of GCs in circulation. Both groups had a decreased expression 11β-HSD2 which converts active GCs to inactive form, more pronounced in Tc-TNF-R1^−/−^ mice.

Corroborating the critical role of TNF-α for host defense, Tc-TNF-R1^−/−^ mice revealed increased parasitemia, accelerated death and more augmented TNF-α, IL-1 and IL-6 concentrations in circulation. Extending these findings, present study suggest that reasons for the accompanying highly increased corticosterone levels may also include processes other than the classical anti-inflammatory reflex resulting from HPA activation. Our data indicate that TNF-α is able to differentially modulate CT secretion at the adrenal level. In situations of a cytokine-mediated intense HPA stimulation at the central level, TNF-α may exert a counterbalancing effect *in situ*, resulting in a highly augmented GCs response and the corresponding adverse effects. Whatever the case, our study adds novel information on the effect of TNF-α on the adrenal gland function during inflammation, particularly within the context of *T. cruzi* infection.
